# Oral delivery of *Mycobacterium bovis* bacillus Calmette-Guérin (BCG) in alginate spheres to captive white-tailed deer

**DOI:** 10.1186/s12917-025-04643-w

**Published:** 2025-03-22

**Authors:** Paola M. Boggiatto, Haley Sterle, Luis Fernandes, Hayden Hamby, Kurt VerCauteren, Abigail Feuka, Henry Campa, Carly Kanipe, Steven C. Olsen, Mitchell V. Palmer

**Affiliations:** 1 Infectious Bacterial Diseases of Livestock Research Unit, National Animal Disease Center, Agricultural Research Service, United States Department of Agriculture, Ames, IA 50010 USA; 2 Immunobiology Graduate Program, Iowa State University, Ames, IA 50010 USA; 3 Oakridge Research Institute for Science Education (ORISE), Oakridge, TN 37830 USA; 4 National Wildlife Research Center, Animal and Plant Health Inspection Service, United States Department of Agriculture, Fort Collins, CO 80521 USA; 5 Department of Fisheries and Wildlife, College of Agriculture and Natural Resources, Michigan State University, East Lansing, MI 48824 USA

**Keywords:** BCG vaccine, White-tailed deer, *Mycobacterium bovis*, T cell, Oral vaccine, Sodium alginate spheres

## Abstract

**Background:**

Bovine tuberculosis (bTB), caused by infection with *Mycobacterium bovis*, continues to be an animal and zoonotic concern in many parts of the world, including the United States. Long-standing eradication programs have been successful at lowering prevalence of disease in many countries; however, disease eradication has not been achieved. One major obstacle to eradication is the presence of various wildlife reservoirs for *M. bovis*, such as white-tailed deer (*Odocoileus virginianus*), which serve as a source of spill-back to cattle herds. A potential method to reduce intra- and inter-species disease transmission of *M. bovis* between wildlife and domestic livestock includes vaccination of wildlife species. Oral vaccination of white-tailed deer with the human tuberculosis vaccine, *M. bovis* bacillus Calmette-Guérin (BCG) has been demonstrated to afford some level of protection against experimental challenge. However, vaccinating wildlife presents its own challenges, primarily due to the need of a delivery platform that could be implemented at scale and would not require animal handling.

**Results:**

Oral vaccine delivery units or baits are an effective means of delivering vaccine to wildlife populations. Therefore, we explored whether sodium alginate spheres could be used as a delivery platform for BCG for vaccination of white-tailed deer. We assessed the development of peripheral immune responses following BCG vaccination and demonstrated that passive administration of BCG via alginate spheres results in antigen-specific cellular responses, similar to oral administration of BCG.

**Conclusions:**

Our data characterize the kinetics of cellular responses elicited by oral vaccination and suggest passive oral administration of BCG as a potential means to vaccinate free-ranging white-tailed deer.

**Supplementary Information:**

The online version contains supplementary material available at 10.1186/s12917-025-04643-w.

## Background

*Mycobacterium bovis* is the causative agent of tuberculosis in many mammals, most importantly, cattle. However, *M. bovis* can cause disease in humans that is clinically indistinguishable from that caused by *Mycobacterium tuberculosis*, the more common cause of human tuberculosis. Concern over potential transmission of *M. bovis* from cattle to humans prompted many countries to implement national programs to eradicate *M. bovis* from cattle. These long-standing and costly campaigns, combined with mandatory pasteurization of milk, have been successful in decreasing the prevalence of *M. bovis* infection in humans and cattle; however, several obstacles have prevented successful eradication in most countries.

One of the most daunting obstacles is the presence of *M. bovis* in wildlife with recurrent wildlife-to-cattle transmission. Several wildlife species are considered reservoir hosts of *M. bovis*; that is, a host population in which *M. bovis* infection can be self-sustaining and permanently maintained. It is generally accepted that wildlife reservoir hosts of *M. bovis* include the brushtail possum (*Trichosurus vulpecula*) in New Zealand, European badger (*Meles meles*) in Great Britain and Ireland, African buffalo (*Syncerus caffer*) in South Africa, wild boar (*Sus scrofa*) and red deer (*Cervus elaphus*) in the Iberian Peninsula, and white-tailed deer (*Odocoileus virginianus*) in the United States [1].

Various approaches have been used to address the problem of wildlife reservoirs of *M. bovis* including the use of poisons in New Zealand to reduce possum numbers [2], population control through increased antlerless deer hunting [3–6], elimination of supplemental feeding and baiting of wildlife [5], or separation of wildlife and cattle through barrier fencing [7–9]. An additional tool that has been investigated in many countries to decrease intra- and inter-species transmission of *M. bovis* is a vaccine for use in wildlife.

Regardless of the wildlife host species, the vaccine most examined has been the human tuberculosis vaccine, *M. bovis* bacillus Calmette-Guérin (BCG) [10–20]. *Mycobacterium bovis* BCG was originally attenuated through repeated serial passage on agar made from potatoes and ox bile. After demonstrating efficacy in animal models including cattle, it was first used in humans in 1921. With over 4 billion doses having been administered, it is the oldest and most widely used vaccine in the world [21, 22]. In animals, BCG has been administered orally, intramuscularly [23, 24] and conjunctivally depending on the wildlife host species of interest [25].

In white-tailed deer, whether administered parenterally or orally, BCG decreases lesion severity [26–30]. Specifically, BCG vaccination decreases the number of highly necrotic lesions, many of which contain large numbers of *M. bovis* bacilli. Highly necrotic lesions are more prone to invade airways and vasculature resulting in bacilli containing respiratory droplets or dissemination of disease, respectively [31]. It is believed that this decrease in disease severity translates into decreased disease transmission, although this remains to be clearly demonstrated. Nevertheless, decreased disease severity has been demonstrated in white-tailed deer when BCG is administered orally as a free liquid followed by experimental challenge with virulent *M. bovis* [17, 26–30].

While oral administration of BCG has shown promise as a possible vaccination strategy, manual oral delivery of the vaccine is not a suitable means for vaccination of free-ranging deer. However, consumption of oral baits is an effective means of delivering vaccine to wildlife populations [32, 33], and thus a desired method for delivering liquid BCG for oral consumption. Furthermore, we sought a delivery platform that would increase residence time of the BCG vaccine in the oral cavity to increase exposure of the oral mucosa. Therefore, we developed a method to encapsulate liquid BCG within a membrane of crosslinked alginate using the reverse spherification technique. When a solution of calcium lactate is added to a bath of sodium alginate, the resulting spherically shaped vessels retain a liquid center and rupture under pressure, coating the inside of the mouth when masticated. By incorporating the encapsulated BCG vaccine in a bait that is known to be desirable to white-tailed deer, a convenient strategy for delivering BCG to white-tailed deer in a field setting emerges.

The objective of this study was to examine if sodium alginate spheres could serve as a successful platform for oral delivery of BCG. To assess their potential, we measured peripheral T-cell proliferative responses in captive white-tailed deer following oral consumption of liquid BCG contained within alginate spheres. The ability to contain liquid BCG within an edible sphere will facilitate the use of BCG in vaccine delivery platforms suitable for use with wild white-tailed deer.

## Results

### T cell subsets following oral vaccination with BCG

**Fig. 1:**
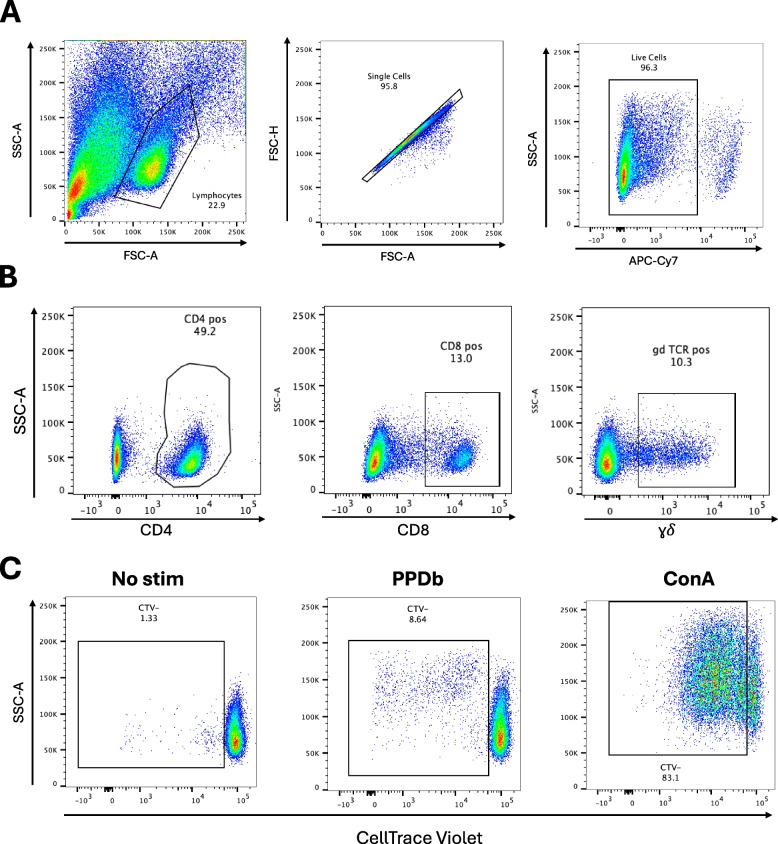
Gating strategy and representative dot plots of flow cytometry data. Representative dot plots of forward scatter (FSC) vs. side scatter (SSC) (first panel), singlet discrimination (second panel), and live-dead determination (last panel) are shown (**A**). Live cells were then gated on CD4 (first panel), CD8 (second panel), and γδ T cells (third panel) (**B**). Each T cell subset was then analyzed for proliferation via CellTrace® violet fluorescence dilution. Shown are representative dot plots for proliferative responses when cells are left unstimulated (No stim; first panel), stimulated with PPDb (second panel), or stimulated with ConA as a positive control (third panel) (**C**)

**Fig. 2:**
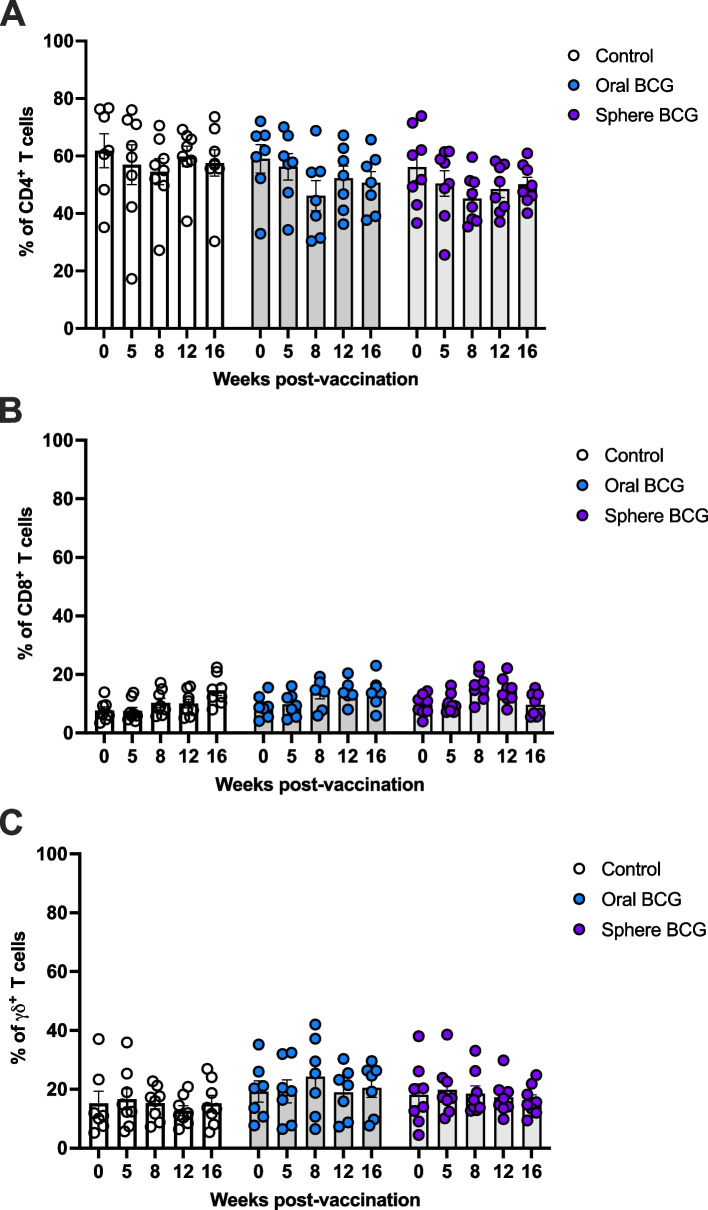
Total frequency of T cell subsets following in vitro antigen stimulation of PBMC from controls and BCG vaccinated animals. PBMC from control (open circles), oral BCG (blue circles), and sphere BCG (purple circles) were stimulated with PPDb antigen in vitro. The total frequencies of CD4 (**A**), CD8 (**B**) and γδ (**C**) T cells were assessed. Shown are mean frequencies for each T cell subset and experimental group. Frequency for individual animals are shown in circles; bars indicate mean frequency for each timepoint; error bars indicate ± SEM

Prior to, and following vaccination, whole blood was collected at various times as indicated above, to assess peripheral cellular mediated immune responses. Cells were first gated on lymphocytes, singlet discrimination, and live cells (Fig. 1A, Supp. Figure 1). Subsequently, live cells were gated on CD4, CD8 and γδ expression (Fig. 1B). Following in vitro stimulation with *M. bovis* purified protein derivative (PPDb), we assessed the overall frequency of CD4 (Fig. 2A), CD8 (Fig. 2B) and γδ (Fig. 2C) T cells. CD4 T cells comprised most of the T cells found in culture, with approximately 60% (56—61%) for control (open circles), oral BCG vaccinated (blue circles) and sphere BCG vaccinated (purple circles) animals (Fig. 2). Frequencies of CD8 T cells ranged from 9–18% (Fig. 2B) and average frequencies of γδ T cells ranged from 15–19% (Fig. 2C), for all experimental groups. Overall, no statistically significant differences were observed in any of the T cell populations between or within vaccinated groups at any of the time points analyzed. These data suggest that in terms of the peripheral response, vaccination with either platform did not affect the overall frequencies of circulating and responding T cells.

### Antigen-specific proliferative responses following oral vaccination with BCG

**Fig. 3:**
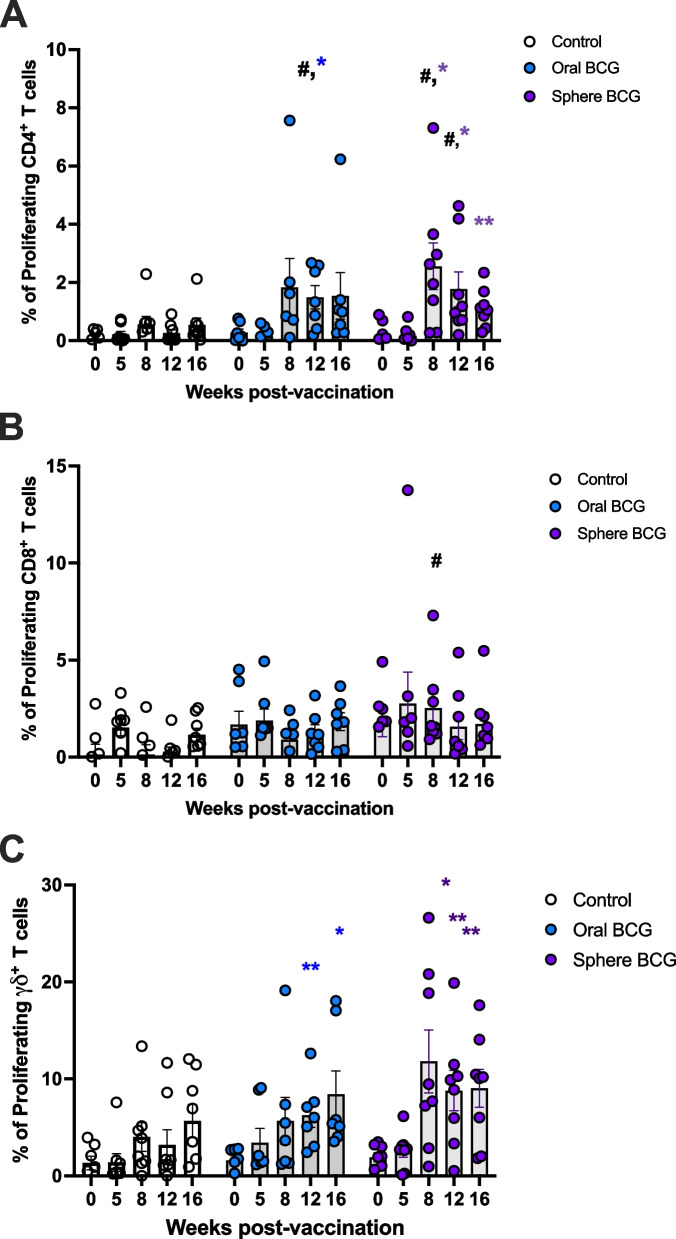
Detectable proliferative responses of T cell subsets following in vitro antigen stimulation of BCG vaccinated animals. PBMC from control (open circle), oral BCG (blue circles), and sphere BCG (purple circles) were stimulated with PPDb antigen in vitro and assessed for proliferation. Shown are the frequency of proliferating CD4 (**A**), CD8 (**B**) and γδ (**C**) T cells. Frequency for individual animals are shown in circles; bars indicate mean frequency for each timepoint; error bars indicate ± SEM. Statistical significance is based on comparisons between and within vaccinate groups (control, oral BCG and sphere BCG). # denotes statistical significance between oral BCG or sphere BCG and control deer, *p* < 0.05. * Denotes statistical significance within groups as compared to pre-vaccination time point, *p* < 0.05. ** Denotes statistical significance within groups as compared to pre-vaccination time point, *p* < 0.001

Each T cell subset was then analyzed for proliferation via fluorescent dye dilution and measured via flow cytometry. Representative dot plots showing proliferation are shown in Fig. 1C. Proliferative responses following stimulation with PPDb were assessed to determine if vaccination resulted in antigen-specific responses. The frequency of proliferating CD4 (Fig. 3A), CD8 (Fig. 3B) and γδ (Fig. 3C) T cells were assessed. Increased frequencies of proliferating CD4 T cells from oral BCG vaccinated deer (blue circles) were observed between 8 – 16 weeks post-vaccination and were statistically different from control deer (open circles) and pre-vaccination frequencies (day 0) at the 12- week timepoint (*p* = 0.021 and *p* = 0.0192, respectively) (Fig. 3A). Deer vaccinated with sphere BCG (purple circles) also demonstrated increased frequencies of proliferating CD4 T cells between 8 – 16 weeks post-vaccination. Proliferative CD4 T cells measured in deer vaccinated with sphere BCG were statistically different from control deer at 8 (*p* = 0.044) and 12 (*p* = 0.039) weeks post-vaccination, and statistically different from pre-vaccination at 8- (*p* = 0.012), 12- (0.020), and 16- (*p* = 0.0019) weeks post-vaccination (Fig. 3A).

When CD8 T cell responses were analyzed, we only observed a statistically significant (*p* = 0.021) increase in the frequency of proliferating CD8 T cells in the sphere BCG vaccinated deer (purple circles) at 8 weeks post-vaccination, as compared to control animals (open circles) (Fig. 3B). Lastly, we analyzed the proliferative response to antigen stimulation within γδ T cells. As compared to control deer (open circles), there was an increasing trend in the frequency of proliferating γδ T cells in both oral BCG (blue circles) and sphere BCG (purple circles) vaccinated deer, but these changes were not statistically significant at any of the time points analyzed (Fig. 3C). However, when compared to pre-vaccination frequencies of proliferating γδ T cells, oral BCG vaccinated deer demonstrated a significant increase in proliferating γδ T cells at 12- (*p* = 0.003) and 16- (*p* = 0.021) weeks post-vaccination. Similarly, the frequency of proliferating γδ T cells was statistically different from pre-vaccination in sphere BCG vaccinated deer at the 8- (*p* = 0.019), 12- (*p* = 0.0098), and 16- (*p* = 0.0058) week timepoints.

Altogether, these data demonstrate measurable T cell proliferative responses following vaccination within CD4 and γδ T cells following vaccination with both oral BCG and sphere BCG. This suggests that both platforms are viable routes for effective delivery of BCG to deer. Additionally, the data indicate that the window for assessment of peripheral cellular responses is found between 8- and 12- weeks post-vaccination.

### Humoral response to BCG vaccination via enzyme linked immunosorbent assay (ELISA)

**Fig. 4:**
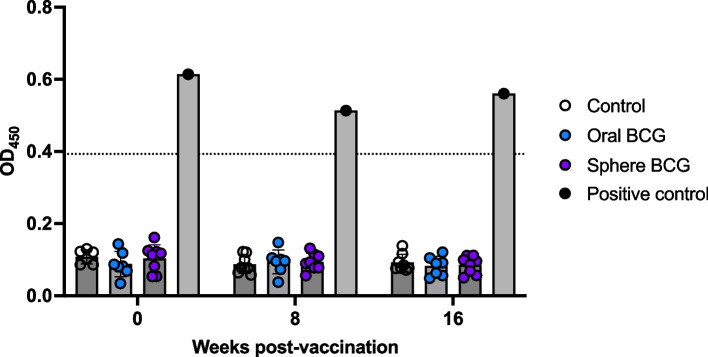
Humoral responses following BCG vaccination. Serum was collected from control (open circles), oral BCG (blue circles) and sphere BCG (purple circles) vaccinated animals and assessed for the presence of IgG antibodies against whole killed *M. bovis* antigens. Shown are mean OD_450_ values for each experimental group. Serum from an *M. bovis* infected white-tailed deer was used as a positive control for the assay. Dotted line indicates assay’s cutoff value for reactors. Frequency for individual animals are shown in circles; bars indicate mean frequency for each timepoint; error bars indicate ± SEM

Humoral responses to vaccination were measured via an in-house whole, killed *M. bovis* lysate ELISA. Following vaccination, we did not observe any increases in OD values in serum samples from either oral BCG or sphere BCG vaccinated animals (Fig. 4), indicating vaccination did not result in measurable IgG responses to mycobacterial antigens at the timepoints analyzed.

## Discussion

In this study, we test the ability of alginate spheres to serve as an oral delivery platform for liquid BCG for use in white-tailed deer. We demonstrate that alginate spheres are a suitable delivery vehicle as shown by the induction of peripheral cellular immune responses to BCG following vaccination. Although we did not challenge these animals to test vaccine efficacy, we do show that both oral BCG and sphere BCG vaccines elicit similar T cell responses; and in both cases, we observed statistically significant different responses as compared to non-vaccinated controls. While cellular immune responses and their components are necessary for protection against TB [34–36], they may not be used as correlates of protection [37, 38]. Cattle vaccinated with BCG develop robust T helper 1 responses, and evidence suggests that proliferating, polyfunctional CD4 T cells expressing interleukin-2 (IL-2), tumor necrosis factor-alpha (TNF-α), and IFN-γ are critical for protection [39]. At this time reagents for evaluating white-tailed deer cells via flow cytometry are limited and only allow us to assess proliferative T cell responses following vaccination. However, we speculate that the presence, similar kinetics, and magnitude of proliferative T cell responses between the two vaccinate groups, suggest that sphere BCG vaccinated deer would exhibit a similar level of protection as oral liquid BCG vaccinated deer. Despite this limitation (*i.e.* the lack of a challenge study) we have previously demonstrated that oral BCG vaccination of white-tailed deer does provide protection against experimental challenge with virulent *M. bovis* [27, 30, 40], characterized by decreased disease severity and decreased bacterial load in the pulmonary lymph nodes of infected deer. Furthermore, protection was achieved when the vaccine was delivered both via direct administration of liquid BCG into the oral cavity or through consumption of lipid-formulated baits [28, 30]. The success of alginate sphere BCG in inducting proliferative responses is promising as a potential candidate for a delivery vehicle for BCG. Nevertheless, vaccine efficacy can only be tested through challenge, and these are ongoing studies in our laboratory.

Reducing deer density and co-mingling, segregation approaches to limit wildlife-livestock interactions, continued surveillance, and education programs have been used in Michigan to control the spread of bTB [41]. While successful in reducing the overall prevalence of bTB in white-tailed deer, these strategies have not resulted in complete control of inter- and intra-species disease spread [6, 41], with continued spill over into cattle herds in endemic areas [42]. Additionally, on-going demographic changes in Michigan including a significant decrease in the number of hunters and a shift in public values regarding human intrusion and/or domination over wildlife, may significantly affect the established bTB surveillance and control program [43]. Efforts are already in place to try to mitigate the effects of these demographic changes, targeting diverse stakeholders, promoting the formation of cooperatives among adjacent landowners to consolidate land and deer management practices, and build long-term trusting relationships between management agencies and the public [43]. However, additional tools to control disease may be necessary, such as the inclusion of vaccines for free-ranging deer.

Managing a susceptible population through immunization or vaccination is a viable approach to disease control; and often, the most cost-effective approach. In the case of free-ranging wildlife species, passive, oral delivery of vaccines would be the most effective method of mass immunization [32, 33]. Orally distributing vaccines has been shown to be effective at reducing and even eliminating disease in free-ranging species, as demonstrated by the success of various rabies vaccination programs [44]. Given the numerous wildlife reservoirs for bTB (*i.e.* brushtail possum, European badger, African buffalo, wild boar, red deer and white-tailed deer), efficacious oral vaccines distributed at scale would be a valuable alternative and/or complementary tool in the arsenal to help control disease spread. Experimental and field studies have demonstrated the safety and efficacy of BCG vaccination against bTB in livestock and wildlife species, albeit with variable rates of efficacy (reviewed in [45, 46]). Recently, a disease transmission model using 10 years’ worth of surveillance data from the four affected counties in Michigan, evaluated the effects of introducing vaccination as a management tool. Using their fitted model, Pandey et al*.* predicted that vaccination of free-ranging white-tailed deer, either as a supplement or alternative management technique, could have a significant impact for disease control. Even with a moderate (60%) vaccine coverage that is  80% effective, significant reductions in disease prevalence could be achieved in six years [47]. In the model, annual vaccination resulted in the highest reduction of prevalence, but biannual and triannual vaccination, also led to a significant reduction in prevalence [47]. Furthermore, as previously shown by others [48], time to bTB elimination can be reduced through the combination of annual vaccinations with increased harvest [47]. These data also highlight that vaccination, as with all other management efforts, would require long-term commitment but would be a valuable tool.

## Conclusions

Our results are the first step in demonstrating alginate sphere technology can be used as an encapsulation method for BCG to white-tailed deer. The spheres allowed for liquid vaccine to be delivered orally to penned animals. BCG-laden spheres will likely be deployed in a food matrix more palatable to free-ranging deer, such as alfalfa [49]. In a recent study, BCG vaccine was directly encapsulated in alginate spheres and encased in such a matrix for deployment in the field. These vaccine delivery units were successfully consumed by free-ranging white-tailed deer in northern Michigan [50], demonstrating this methodology has potential for operational use. However, more laboratory studies will need to be conducted to confirm that BCG-laden spheres consumed within a food matrix still produce an immunological response and provide adequate protection when challenged with *M. bovis.* Furthermore, studies looking at the viability/stability of BCG within alginate spheres, in liquid form, are needed to understand the advantages and limitations of this vaccination format. While these studies are beyond the scope of this manuscript, the work presented here lays the foundation for further assessment of how environmental conditions could affect this platform.

## Methods

### Vaccine preparation and alginate sphere production

The BCG Danish vaccine was grown in Middlebrook’s 7H9 media supplemented with 10% oleic acid-albumin-dextrose catalase (OADC) (Difco, Detroit, MI) plus 0.05% Tween 80 (Sigma Chemical Co., St. Louis, MO) as described [51, 52]. Mid log-phase growth bacilli were pelleted by centrifugation at 750 × g, washed twice in PBS (0.01 M, pH 7.2) and stored at −80 °C until used. Prior to use, frozen stock was warmed to room temperature (RT) and diluted to the appropriate cell density in PBS. Bacilli were enumerated by serial dilution plate counting on Middlebrook’s 7H11 selective media (Becton Dickinson, Cockeysville, MD, USA).

**Fig. 5:**
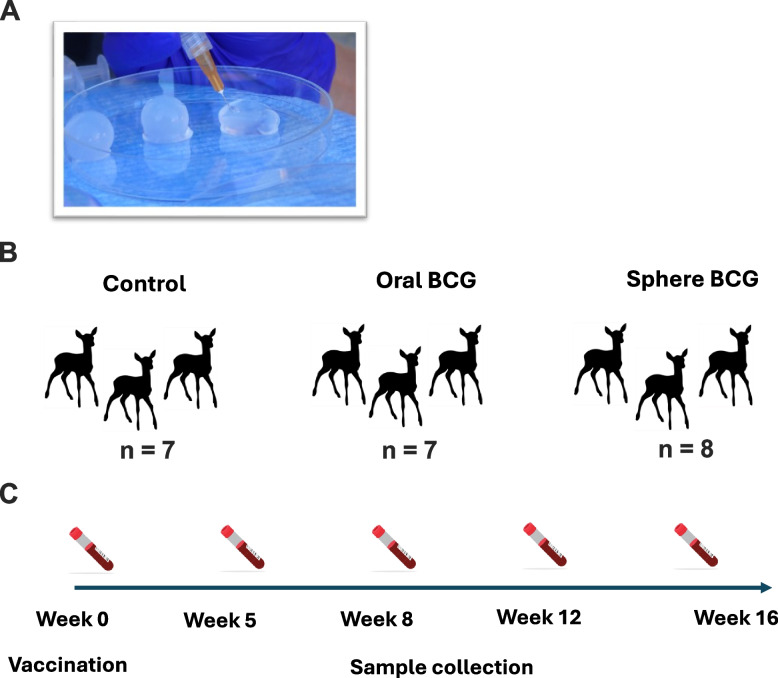
Alginate spheres and experimental design. Alginate spheres were approximately 1–2 cm in size, are shown being filled with BCG prior to vaccination (**A**). Experimental groups (**B**) and time points for vaccination and blood sample collection (**C**) are shown

Alginate spheres (Fig. 5A) were generated using a mixture of a calcium solution and an alginic acid solution. For the calcium solution, 200 g (gm) water (18.2 MΩ·cm resistivity and < 10 parts per billion (ppb) total organic carbon) were added to a 250 ml glass beaker followed by 4.40 gm calcium lactate pentahydrate (Fisher Scientific, USP, ≥ 98%), and 10 gm of sugar (C&H Sugar Co., granulated white). This mixture was blended until homogenous using a Polytron PT 10–35 equipped with a Kinematica AG PTA 10TS Aggregate 12-mm (mm) head at setting #4 for 30 s. Next, 2.00 gm of xanthan gum (Gum Tech. Coyote Brand, 80 mesh) were added, and the mixture blended an additional 30 s until smooth.

For the alginic acid solution, 1000 gm of water, 50 gm of sugar, and 10.00 gm of alginic acid salt (Thermo Scientific, low viscosity, ≤ 13% w/w loss on drying) were added to a Waring Commercial Heavy-Duty Blender and blended on the lowest setting in 10 s intervals. Between blending intervals, the material was scraped from the container walls down into the solution and the process repeated until homogeneous. Prior to use, air bubbles were removed by centrifugation or by allowing the solution to rest in an observation dish at room temperature (RT) for at least 30 min.

To form the spheres, the alginic acid solution was poured into a glass observation dish with the following dimensions 165 mm diameter and 85 mm height. Aliquots of the calcium solution (2 – 2.2 ml) are dispensed into the bath of the alginic acid solution using a 5 ml pipettor equipped with a modified tip such that the opening is 6 mm in diameter. The reaction was allowed to proceed for approximately 10 min before removal and immersion in a water bath. All spheres were made at the National Wildlife Research Center in Fort Collins, Colorado, USA and shipped overnight in water to Ames, IA, USA for trials. On arrival, spheres were stored in water at 4° C until use, for approximately 1 week. Immediately prior to administering to deer, the contents of the alginate spheres were removed and replaced with 2.0 ml BCG in PBS. Spheres were approximately 1 – 1.6 cm in diameter.

### Animal vaccination

Twenty-two white-tailed deer (~ 1 yr-old) were obtained from a tuberculosis free captive breeding herd at the National Animal Disease Center (NADC) in Ames, IA, USA. This study was carried out in strict accordance with the Guide for the Care and Use of Laboratory Animal of the National Institutes of Health (NIH) and the Guide for the Care and Use of Agricultural Animals in Agricultural Research and Teaching of the Federation of Animal Science Societies. The NADC Institutional Animal Care and Use Committee (IACUC) approved protocols prior to implementation (Protocol number ARS-23–1127). All deer used in this study were housed outdoors. Vaccinations were performed in late summer/early fall.

Deer were randomly assigned using a random number generator to one of three groups: orally vaccinated with 1 × 10^8^ colony-forming units (CFU) of *M. bovis* BCG Danish in 2.0 ml phosphate buffered saline (PBS) (*n* = 7); orally vaccinated with 1 × 10^8^ CFU M*. bovis* BCG Danish in 2.0 ml PBS contained within an alginate sphere (*n* = 8); and non-vaccinated (*n* = 7) (Fig. 5B). Deer vaccinated with liquid BCG were vaccinated as previously described [30]. Briefly, with the aid of a swine mouth speculum, BCG suspended in 2.0 ml PBS was administered to the posterior pharynx using a 3 ml syringe and a 10-French (0.33 mm) 25 cm sterile urinary catheter (Monoject, St. Louis, MO, USA). Vaccine-containing spheres were delivered to the oral cavity (one sphere per animal) and the deer allowed to chew and consume the sphere. Non-vaccinated deer received no vaccine.

Deer were housed separately according to treatment group for one month prior to recombining groups to avoid vaccine shedding and transmission, a phenomenon that has been seen in white-tailed deer vaccinated with BCG strain Pasteur, but not BCG strain Danish [26, 27].

### Peripheral blood mononuclear cell (PBMC) isolation

Blood samples were collected via jugular vein venipuncture prior to (week 0) and at 5-, 8-, 12-, and 16-weeks following vaccination to assess peripheral T cells responses to the vaccine (Fig. 1C). Blood was collected into EDTA tubes and PBMC were isolated via Ficoll (1.077) gradient centrifugation as previously described [53]. Cellular viability and counts were obtained using the Muse™ Cell analyzer (Cytek, Fremont, CA). Cell suspensions were then adjusted to 1 × 10^7^ viable cells per milliliter (ml) in Dublecco’s phosphate buffered saline (DPBS).

### In vitro recall responses

To assess proliferation of T cells, PBMC were stained with CellTrace Violet (CTV; Invitrogen) as previously described [53]. Stained cells were then plated onto 96-flat bottom plates at a density of 1 × 10^6^ PBMC per well in complete RPMI (cRPMI). Cells were then stimulated with purified protein derivative (PPDb, 30 ug/well, National Veterinary Services Laboratories, Veterinary Services, Ames, IA), Concanavalin A (ConA) (0.5 ug/well) (Sigma), or left unstimulated in media alone for 7 days at 37 °C with 5% CO_2_. All stimulation conditions were plated in duplicate.

### Surface staining and flow cytometry analysis

At the end of the incubation period, cells were harvested, and duplicate wells combined and replated onto 96-well round bottom plates. Cells were then washed with DPBS at 300 × g for 5 min (min) at RT. Cells were stained with a cell viability dye (Invitrogen) and then prepared for surface staining using antibodies against CD4, CD8 and γδ, as described previously [53]. Briefly, cells were washed in FACS buffer (0.5% FBS in PBS) at 300 × g for 5 min at RT, and then incubated with primary antibodies against goat CD4 (clone: 17D; IgG1), goat CD8 (clone: ST8, IgM), and bovine γδ (clone: GB21A, IgG2b) (Washington State) at a 1:100 dilution for 15 min at RT. Cells were then incubated with the following secondary antibodies, BUV395-labeled anti-IgG1 (BD Bioscience, San Diego, CA), FITC-labeled anti-IgM (Biolegend, San Diego, CA), and BV711-labeled anti-IgG2b (BD Bioscience) at a 1:100 dilution for 15 min at RT. Cells were then washed twice in FACS buffer and fixed in BD Stabilizing Fixative (BD Bioscience). Data was acquired using FACS Symphony A5 (BD Bioscience) flow cytometer.

### Enzyme linked immunosorbent assay (ELISA)

Blood samples were collected via jugular vein venipuncture prior to (week 0) and at 5-, 8-, 12-, and 16-weeks following vaccination and placed into serum top tubes and allowed to clot at RT. Tubes were then centrifuged at 800 × g for 30 min and serum was aliquoted and stored at −80 °C until analysis. To evaluate the functionality of secondary anti-goat IgG against white-tailed deer antibodies, ELISA plates (Costar, high binding) were coated in triplicate with 50 µL of either deer or goat serum. After washing and blocking with SuperBlock (Thermo Fisher), the wells were incubated with horseradish peroxidase (HRP)-conjugated anti-goat IgG (Jackson ImmunoResearch) at a 1:10,000 dilution in SuperBlock buffer. Reactivity was detected by adding 100 µL of KPL SuperBlue TMB substrate (SeraCare) to each well. After a 10-min incubation, the reactions were stopped with 100 µL of KPL TMB BlueSTOP solution (SeraCare), and color development was measured at 650 nm using an ELISA plate reader (BioTek Synergy Neo2, Agilent, Fisher Scientific).

To detect IgG antibodies in deer serum samples following BCG vaccination, ELISA plates were coated with 100 µL of a BCG cell lysate containing 10⁸ cells/mL and incubated overnight at 4 °C. To eliminate cross-reactivity with *Mycobacterium avium* subspecies *paratuberculosis* (MAP), serum samples were diluted 1:50 in SuperBlock buffer containing MAP cell lysate (10⁸ cells/mL) and incubated overnight at 4 °C prior to exposure to the coated BCG antigens. The ELISA plates were then washed three times with PBS containing 0.05% Tween-20 (PBS-T) and blocked for 1 h with 250 µL of SuperBlock buffer. After discarding the blocking solution, 100 µL of the MAP-adsorbed serum was added to each well in triplicate and incubated for 1 h at 30 °C. A positive control consisting of an in-house BCG-reactive deer serum sample was included in all experiments. Following incubation, the wells were washed three times, and 100 µL of HRP-conjugated anti-goat IgG (1:10,000) were added. This was followed by a 1 h incubation and six subsequent washing steps with PBS-T and reactivity was developed as described previously. Blank controls, to which no serum was added, were included in all experiments. The cutoff value for positivity was determined as the mean absorbance plus three standard deviations of the absorbance for serum samples obtained before BCG vaccination.

### Statistical analysis

The frequency of total and proliferating T cell subsets (CD4, CD8, and γδ) were determined using FlowJo™ software (FlowJo, LLC, BD Bioscience, Ashland, OR). Frequencies reported for proliferation in response to PPDb stimulation were normalized by removing the background proliferation observed in unstimulated cells. The data were graphed and analyzed using GraphPad Prism 9 (Graphpad software, Boston, MA). Changes over time were evaluated by repeated measures with a mixed model. Specific contrasts of interest, both within group (compared to pre-vaccination (day 0)) and between vaccinate groups at all time points, were evaluated with an ANOVA fitting vaccination status (control, oral BCG, or sphere BCG), time (weeks post-vaccination), and a (vaccination x time) interaction. Significance was determined when *p*-value ≤ 0.05; error bars represent standard errors.

## Supplementary Information

Supplementary Material 1: Supplementary Figure 1. Representative dot plots of FSC vs. SSC with and without PPDb stimulation. Shown are representative dot plots showing FSC vs. SSC of control (left panels), BCG vaccinated (middle panels), and sphere BCG vaccinated (right panels) animals. Top panels show PBMC left unstimulated and bottom panels show PMBC stimulated with PPDb.

Supplementary Material 2: Supplementary Data File 1. Table containing all flow cytometry data used in these studies.

## Data Availability

All data generated or analyzed during this study are included in this published article and its supplementary information files (Supplementary Data File 1).
