# Supplementary figures and images for: Oral delivery of Mycobacterium bovis bacillus Calmette-Guérin (BCG) in alginate spheres to captive white-tailed deer
Source: BMC Vet Res. 2025 Mar 22;21:193. doi: 10.1186/s12917-025-04643-w (PMC11929337; doi:10.1186/s12917-025-04643-w)

Control

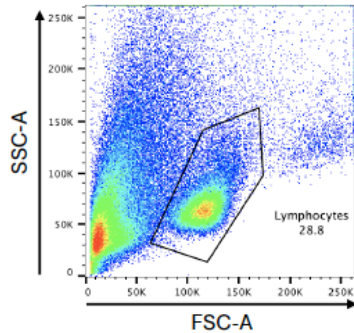

BCG Vax

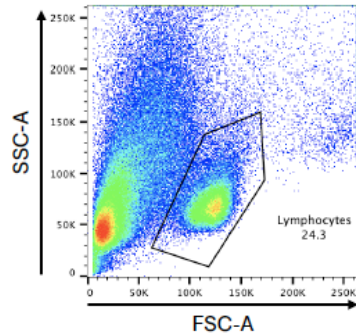

Sphere Vax

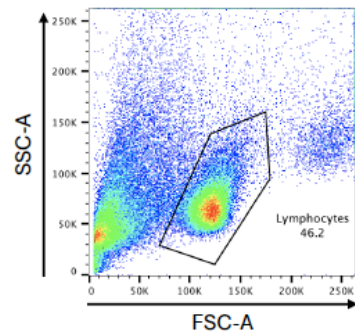

PPDb Stim

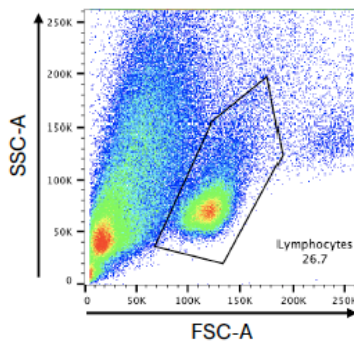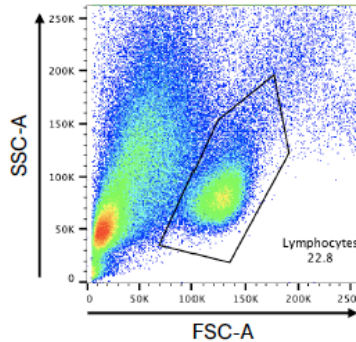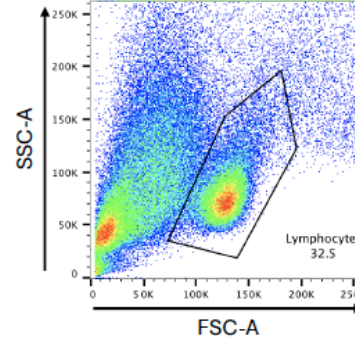

Supplement: Supplementary file 1 — Supplementary Material 1: Supplementary Figure 1. Representative dot plots of FSC vs. SSC with and without PPDb stimulation. Shown are representative dot plots showing FSC vs. SSC of control (left panels), BCG vaccinated (middle panels), and sphere BCG vaccinated (right panels) animals. Top panels show PBMC left unstimulated and bottom panels show PMBC stimulated with PPDb. [file 12917_2025_4643_MOESM1_ESM.pdf]
